# Assessing the Causal Role of Selenium in Amyotrophic Lateral Sclerosis: A Mendelian Randomization Study

**DOI:** 10.3389/fgene.2021.724903

**Published:** 2021-10-06

**Authors:** Di He, Liying Cui

**Affiliations:** ^1^ Department of Neurology, Peking Union Medical College Hospital (PUMCH), Beijing, China; ^2^ Chinese Academy of Medical Sciences and Peking Union Medical College (CAMS and PUMC), Beijing, China

**Keywords:** amyotrophic lateral sclerosis, mendelian randomization analysis, selenium, environmental factor, epidemiology

## Abstract

**Objectives:** The relation between selenium overexposure and increased risk of amyotrophic lateral sclerosis (ALS) has been subject to considerable interest. Epidemiologic studies have reported suggestive associations between selenium and ALS, although the causal inference between selenium and ALS remains to be established.

**Methods:** We conducted a two-sample Mendelian randomization (MR) analysis to analyze the causal role of selenium on ALS risk. Variants associated with selenium levels were obtained from the GWAS meta-analysis of circulating selenium levels (*n* = 5,477) and toenail selenium levels (*n* = 4,162) in the European population. Outcome data were from the largest ALS GWAS dataset with 20,806 ALS cases and 59,804 controls in the European population. Inverse variance weighted (IVW) method was used as the main analysis, with an array of sensitivity analyses performed to detect potential violations of MR assumptions.

**Results:** Inverse variance weighted (IVW) analysis indicated no evidence of a causal role for selenium levels in ALS development (odds ratio (OR) = 1.02, 95% confidence interval (CI) = 0.96–1.08). Similar results were observed for the sensitivity analyses (OR = 1.00, 95% CI = 0.95–1.07 for weighted median; OR = 1.07, 95% CI = 0.87–1.32 for MR-Egger), with no pleiotropy detected.

**Conclusions:** Although selenium was found associated with ALS according to earlier epidemiologic studies, current evidence based on the population of European ancestry does not support the causal effect of selenium on ALS risk.

## Introduction

Amyotrophic lateral sclerosis (ALS) is a paralytic disorder progressively affecting both upper and lower motor neurons (Swinnen and Robberecht, 2014; Brown and Al-Chalabi, 2017). It is considered a complex genetic disease, with Mendelian inheritance pattern observed only in a small number of familial cases (Hardiman et al., 2017; Chia et al., 2018). Although multiple ALS risk variants have been identified during the past 2 decades, likely due to incomplete penetrance, these implicated genotypes do not necessarily lead to disease phenotypes (Al-Chalabi et al., 2017; van Es et al., 2017). Alternatively, it has also been suggested that the manifestation of ALS is a stepwise process, in which the predisposing variants carried by individuals interact with multiple environmental triggers (Al-Chalabi et al., 2014; Cook and Petrucelli, 2019). Such multistep model empathizes the relevance of studying both genetic and environmental risk factors in ALS (Paez-Colasante et al., 2015).

Among these environmental factors, studies in the past decades have highlighted the potential role of ionic homeostasis in the etiopathogenesis of ALS (Sirabella et al., 2018; Tesauro et al., 2021). In particular, suggestive epidemiologic evidence seems to support an association between increased ALS incidence and selenium exposure (Vinceti et al., 2012; Vinceti et al., 2013; Filippini et al., 2020). Such relation is further supported by evidence from biological research that certain selenium species may be detrimental to neurons *via* inducing apoptotic process (Xiao et al., 2006; Wandt et al., 2021), which is the pathological feature of ALS. However, although the etiological role of selenium has been frequently investigated, to what extent the pathogenesis of ALS can be ascribed to its exposure remains inconclusive (Longinetti and Fang, 2019). In retrospective studies, the concentrations of suspected risk factors are usually measured after disease onset, which limits their potential to rule out reverse causality, as the damage might have taken place years before the onset, and the observed differences might be the consequence of disease progression. The questionnaire-based observational studies, which rely on self-reported information for the assessment of exposures, are subject to recall and selection biases (Filippini et al., 2020). The prospective case–control study design, on the other hand, is usually restricted by the modest number of cases enrolled, partly due to the low prevalence of ALS in the general population (Peters et al., 2020). Therefore, given the rarity of the disease and ethical issues, it is difficult to conduct unbiased association studies of ALS and selenium in practice.

Two-sample Mendelian randomization (MR) analysis offers us the unique alternative to probe the question of causality via exploiting the massive wealth of the ever-growing number of Genome-Wide Association Studies (GWAS). Analogous to the randomized controlled trial (RCT), two-sample MR uses genetic variants as unbiased proxies for random assignment, thereby enabling us to estimate the causal effect of exposures on the outcome of interest (Smith and Hemani, 2014). Two-sample MR is based on the natural genetic variation effect sizes on the exposure cohort and the outcome cohort, the statistics of which can be derived from their respective summarized GWAS dataset. If the exposure influences the outcome, then the influence of these valid genetic proxies on the outcome is proportional to their effect on the exposure. Since genetic variants are fixed at conception and temporally precede the outcome, MR is less likely biased by reverse causation and confounding (Emdin et al., 2017). In the present study, we evaluated the causal effects of selenium exposure on ALS risk by conducting a two-sample MR analysis with publicly available GWAS summary statistics.

## Materials and Methods

### Exposure Dataset and Genetic Instruments

Summary statics for the genetic variants showing genome-wide significant association (*p* < 5 × 10^–8^) with selenium levels were obtained from the GWAS meta-analysis of circulating selenium levels (*n* = 5,477) and toenail selenium levels (*n* = 4,162) in European-ancestry individuals (Evans et al., 2013; Cornelis et al., 2015). Of note, since the units of toenail and blood selenium level were not comparable, the Z-score were translated from β (SE) for the analysis. The variants were clumped based on 1,000 Genomes Project linkage disequilibrium (LD) structure (*R* ^2^ < 0.3 with any other associated SNP within 10 Mb) to ensure that the selected instrumental variables (IVs) were independently predicting the exposure. The proportion of phenotypic variance explained (PVE) by IVs as well as the F statistics were calculated to test the strength of the instruments.

### Outcome Dataset

The largest publicly available GWAS summary statics for ALS involving 20,806 ALS cases and 59,804 controls of European ancestry was used as outcome data (Nicolas et al., 2018), which was comparable with the exposure dataset given the composition of population ethnicity. The analyses were restricted to ethnically homogeneous group to avoid population stratification (Burgess et al., 2017). Harmonization step was undertaken for effect allele alignment and ruling out strand mismatches (Hartwig et al., 2016) using the TwoSampleMR package. Since only summarized statistics from publicly available GWAS was used, and no individual-level data was involved, ethical approval was not sought for the present study.

### Statistical Analysis

To estimate the causal effect of selenium exposure on ALS, individual Wald-type ratios for each of the IVs were meta-analyzed using the inverse variance weighted (IVW) approach, with Cochran’s Q statistic calculated for heterogeneity. Additionally, extensive sensitivity tests were performed to guard against potential violation of the model assumptions in the MR analysis. Specifically, because the IVW estimate is not guarded against any SNPs violating the IV assumptions, weighted median method, which only requires the majority of variants being valid instruments, was included as a complementary test (Bowden et al., 2016; Bowden et al., 2017), whereas MR-Egger regression was performed to account for the bias caused by directional horizontal pleiotropy (Burgess and Thompson, 2017). Outliers substantially influence causal effect were checked by leave-one-out (LOO) analysis and MR Pleiotropy RESidual Sum and Outlier (MR-PRESSO) (Burgess et al., 2017). Recent advances in statistic methodology have also introduced several novel MR methods dealing with pleiotropy issues, which include MR-Mix (Qi and Chatterjee, 2019), MR-ContMix (Burgess et al., 2020), CAUSE (Morrison et al., 2020), and cML-MA (Xue et al., 2021). Since MR-Mix, MR-ContMix, and CAUSE impose a normal mixture model with various unknown parameters, which makes them technically challenging to perform when only a handful of IVs are available and prone to inflated type I errors, it is not preferable to choose these methods for the present study. Therefore, the method based on constrained maximum likelihood and model averaging (cML-MA) and Bayesian information criterion (BIC) approach was chosen as one of the sensitivity tests to address the issue of potential violation of IV assumptions. Given the small number of available IVs, data perturbation (DP) approach was also adopted as described by Xue et al. (2021). A description of the MR methods used in this study with their respective advantages and limitations can be found in Table 1. Notably, because the summary statistics for selenium variants were expressed in Z-score units per allele (Cornelis et al., 2015), which were converted from beta and standard error values for the purpose of MR analysis, neither the effect sizes from the MR analysis nor the beta values for associations of SNPs with selenium levels have interpretable units. All statistical analyses were conducted using the R package TwoSampleMR (version 0.4.26) and MR-cML.

**TABLE 1:** Mendelian randomization methods used in this study for assessing the association between selenium levels and the risk in developing amyotrophic lateral sclerosis (ALS).

Method	Assumptions	Advantages and limitations	*p*-Value
IVW	Uncorrelated SNP-exposure and SNP-outcome association estimates; NOME	Highest statistical power when all assumptions are met; sensitive to directional horizontal pleiotropy	0.61
Weighted-median	Majority valid	Avoid the contribution of some invalid IVs; low power; potentially biased	0.88
MR-Egger	InSIDE	Allowing a non-zero intercept in cases of directional pleiotropic effects; dramatically lower statistical power than IVW; more susceptible to regression dilution bias	0.52
MR-PRESSO	Majority valid; InSIDE	Reducing IV heterogeneity by outlier removal; inflated type I error rates; potentially biased selection of invalid IVs	0.12
cML-MA-BIC (cML-MA-BIC-DP)	Plurality valid	Sufficiently powered with controlled type I errors. (Better controlled type I errors but lower power than cML-MA-BIC)	0.87 (0.83)

The method assumptions, advantages/limitations, and their respective *p*-value results were as displayed. NOME, no measurement error; InSIDE, instrument strength independent of direct effect.

## Results

**FIGURE 1:**
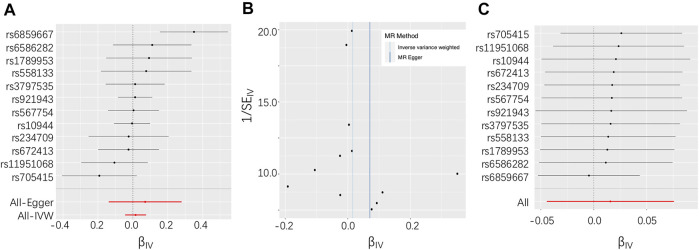
**(A)** Forest plot for individual causal effect estimate based on the set of 12 instruments. **(B)** Funnel plot displaying individual causal effect estimates for selenium on amyotrophic lateral sclerosis (ALS). **(C)** Leave-one-out (LOO) results of selenium on ALS.

In total, 12 independent SNPs, all of which were available in the exposure and outcome datasets, were selected as IVs (Table 2). No heterogeneity of effects was detected using Cochran’s Q test (*p* = 0.08). The genetic instruments explained 0.32–1.76% of the variation in circulating and toenail selenium levels, and the F statistics were larger than 10 for all included IVs, which indicate that the instruments used in the MR analysis were unlikely to suffer from weak instrument bias. The MR analysis did not support an association between selenium levels and ALS risk using the IVW method (OR = 1.02, 95% CI = 0.96–1.08) (Figure 1A). Association estimates from sensitivity analyses such as weighted median, MR-Egger, MR-PRESSO, and cML-MA were consistent with that reported by IVW analysis, as summarized in Table 1. The robustness of the results was confirmed by these sensitivity tests. The test for directional pleiotropy by MR-Egger did not give evidence for pleiotropy in the causality investigated as the intercept did not differ from zero (*p* = 0.60). This was supported by the funnel plot, which displayed symmetric pattern of effect size variation around the point estimate (Figure 1B). The MR-PRESSO analysis detected no potential instrumental outlier (*p* = 0.12), and the LOO analysis also suggested that no single instrumental variable could disproportionally influence the estimated causal effect (Figure 1C).

**TABLE 2:** Characteristics of the instrumental variables used in two-sample Mendelian randomization (MR) analysis.

Instrumental variables	Position (GRCh38.p13)	EA	EAF	Association with the exposure			Association with the outcome	
				Z-score	β (S.E.)	*p*-Value	β (S.E.)	*p*-Value
rs672413	5:78982406	A	0.32	7.53	0.16 (0.02)	5.21E−14	0.00 (0.01)	0.78
rs705415	5:78996137	T	0.14	−6.23	−0.20 (0.03)	4.64E−10	0.04 (0.02)	0.08
rs3797535	5:79004574	T	0.08	7.94	0.30 (0.04)	2.05E−15	0.00 (0.03)	0.88
rs11951068	5:79008491	A	0.07	6.72	0.27 (0.04)	1.86E−11	−0.03 (0.03)	0.28
rs921943	5:79020653	T	0.29	13.14	0.29 (0.02)	1.90E−39	0.00 (0.01)	0.80
rs10944	5:79090022	T	0.49	12.65	0.26 (0.02)	1.13E−36	0.00 (0.01)	0.93
rs567754	5:79120593	T	0.34	−9.11	−0.20 (0.02)	8.38E−20	0.00 (0.01)	0.96
rs558133	5:79129365	A	0.69	−6.55	−0.14 (0.02)	5.60E−12	−0.01 (0.02)	0.56
rs6859667	5:79449219	T	0.96	−6.92	−0.36 (0.05)	4.40E−12	−0.13 (0.04)	4.53E−04
rs6586282	21:43058387	T	0.17	−5.89	−0.16 (0.03)	3.96E−09	−0.02 (0.02)	0.33
rs1789953	21:43062826	T	0.14	5.52	0.16 (0.03)	3.40E−08	0.02 (0.02)	0.46
rs234709	21:43066854	T	0.45	−5.84	−0.12 (0.02)	5.23E−09	0.00 (0.01)	0.84

EA, effect allele; EAF, effect allele frequency; β, per allele effect on the exposure; SE, standard error; *p*-Value, *p*-value for the genetic association.

## Discussion

Since metal homeostasis is critical for normal brain function, an excess of metal levels has been postulated as potential risk factors for a variety of neurodegenerative disorders (Grochowski et al., 2019). Accordantly, the concentration of trace metals in the hair and nails of patients have been found related to the clinical course of Alzheimer’s disease (Koseoglu et al., 2017), and the neurotoxic effects of excess selenium exposure may contribute to ALS etiology according to previous epidemiology studies (Vinceti et al., 2010; Vinceti et al., 2016). It has been noted that the concentration of selenium in urine and scalp hair was elevated in men, which is consistent with the epidemiologic findings that ALS is more common in men than in women (Berglund et al., 2011; Tamburoo et al., 2016; van Es et al., 2017). The neurotoxic effects of selenium might be mediated by inducing oxidation of thiol-containing protein and promoting translocation of copper/zinc superoxide dismutase (SOD1) into mitochondria (Filippini et al., 2018). However, observational studies are prone to reverse causation and various confounders, in which case incorrect causal inference might be made even with careful study design and proper statistical adjustment (Frikke-Schmidt et al., 2008; The Emerging Risk Factors Collaboration*, 2009; Voight et al., 2012). The biomarkers currently used to assess selenium exposure also have inherent limitations, and the reliability of these methods in reflecting the long-term cumulative exposure of selenium has been debated and challenged (Vinceti et al., 2018). In addition, peripheral indicators of selenium exposure may not necessarily correspond to its CNS content, given the independence of selenium level in paired serum and cerebrospinal fluid (CSF) samples (Michalke et al., 2009).

Here we leveraged the summary statistics from recent large-scale GWAS datasets to probe the association between selenium exposure and the risk for ALS. Since two-sample MR assumes that the genetic variants used as IVs influence the outcome because the hypothesized exposure does (vertical pleiotropy), three assumptions need to be satisfied in a valid MR analysis: the selected SNPs are associated with the exposure (the relevance assumption); they are not associated with any confounders (the independence assumption); and they influence the risk of ALS only through the pathway of the exposure (the exclusion assumption) (Greenland, 2000; Emdin et al., 2017). Thus, to validate the IV assumptions, two alternative mechanisms need to be ruled out: IVs also being in LD with a causal variant for the outcome; IVs influencing the outcome through a pathway other than the exposure (horizontal pleiotropy) (Hemani et al., 2018). After LD-based clumping and pruning, multiple independent genetic variants reaching conventional genome-wide significance level (thereby validating the relevance assumption) were meta-analyzed via IVW for an overall estimate of their effect on the outcome in our study. However, although using multiple genetic variants can enhance the statistical power of the MR analysis, the causal estimate would be liable to bias with inflated type I error rates if invalid IVs are included (Burgess et al., 2017). Thus, no variant having potential pleiotropic associations with ALS (defined by an ALS association *p*-value below the genome-wide suggestive significance level of 10^–5^) was included as IV in the current MR analysis. Since the second and third IV assumptions are not fully testable in practice, we compared the estimates from a range of sensitivity analyses (Table 1). Specifically, MR-Egger was applied to assess the uncorrelated pleiotropic effects engendered by the potential violation of the second assumption. Weighted-median and MR-PRESSO were adopted to address potentially invalid IVs. cML-MA, which is robust against both correlated and uncorrelated pleiotropic effects, was used to validate the second and third assumptions. The results of these sensitivity analyses were in accordance with the IVW result. Therefore, the present study did not find evidence supporting any causal relationship between ALS and selenium, which is in accordance with the null association found between ALS and erythrocyte-bound selenium level in a recent prospective case-control study (Peters et al., 2020).

The study is subject to several limitations. First, although there is evidence supporting the existence of variation in the concentration of metals/metalloids by age and gender (Berglund et al., 2011), we cannot decide whether there is any age- or gender-specific effect of selenium exposure on ALS, as individual-level GWAS datasets are not accessible. Second, to avoid population stratification, we focused on subjects of European ethnicity. Whether the findings may be extended to other populations remains unclear. Third, although the correlation between serum and nail content of selenium has been reported to be weaker than between serum and hair (Chawla et al., 2020), conducting two separate MR analyses using their respective GWAS (Evans et al., 2013; Cornelis et al., 2015), both of which were modest in scale, would unavoidably reduce the number of qualifying IVs for the analysis, subsequently limit the statistical power for identifying any hypothesized association. Thus, the 12 genetic variants robustly associated with selenium concentration in the meta-analysis of blood and toenail selenium GWAS were selected as proxies for selenium level. It is also noteworthy that a similar approach has been adopted by other researchers when conducting MR analysis (Yarmolinsky et al., 2018). Finally, although the MR-Egger regression results did not support horizontal pleiotropy, it is difficult to completely rule out pleiotropy or alternative causal pathways in MR analyses. In addition, the MR analysis assumed linearity and homogeneity between the exposure, the genetic variants, and the risk for ALS, which may not represent the true associations in nature. This could potentially limit us from identifying putative thresholds of exposure above or below which the exposure can induce specific effects.

In conclusion, using summary statistics from GWAS, we did not find strong evidence for the causal inference of selenium on the risk of ALS in the present study. Such findings might be informative for epidemiologic studies of ALS in the future.

## Data Availability

Publicly available datasets were analyzed in this study. This data can be found here: https://pubmed.ncbi.nlm.nih.gov/25343990/ https://pubmed.ncbi.nlm.nih.gov/23720494/ https://pubmed.ncbi.nlm.nih.gov/29566793/.
